# Draft genome of a novel methanotrophic *Methylobacter* sp. from the volcanic soils of Pantelleria Island

**DOI:** 10.1007/s10482-021-01525-7

**Published:** 2021-02-10

**Authors:** Carmen Hogendoorn, Nunzia Picone, Femke van Hout, Sophie Vijverberg, Lianna Poghosyan, Theo A. van Alen, Jeroen Frank, Arjan Pol, Antonia L. Gagliano, Mike S. M. Jetten, Walter D’Alessandro, Paola Quatrini, Huub J. M. Op den Camp

**Affiliations:** 1 Department of Microbiology, IWWR, Radboud University, Heyendaalseweg 135, 6525 AJ Nijmegen, The Netherlands; 2 Istituto Nazionale di Geofisica e Vulcanologia, Sezione di Palerma, Via U. La Malfa 153, 90146 Palermo, Italy; 3 Department of Biological, Chemical and Pharmaceutical Sciences and Technologies (STEBICEF), University of Palermo, Viale delle Scienze Ed. 16, 90128 Palermo, Italy

**Keywords:** Methane, Methanotroph, Volcanic soil, Metabolic potential

## Abstract

The genus *Methylobacter* is considered an important and often dominant group of aerobic methane-oxidizing bacteria in many oxic ecosystems, where members of this genus contribute to the reduction of CH_4_ emissions. Metagenomic studies of the upper oxic layers of geothermal soils of the Favara Grande, Pantelleria, Italy, revealed the presence of various methane-oxidizing bacteria, and resulted in a near complete metagenome assembled genome (MAG) of an aerobic methanotroph, which was classified as a *Methylobacter* species. In this study, the *Methylobacter* sp. B2 MAG was used to investigate its metabolic potential and phylogenetic affiliation. The MAG has a size of 4,086,539 bp, consists of 134 contigs and 3955 genes were found, of which 3902 were protein coding genes. All genes for CH_4_ oxidation to CO_2_ were detected, including *pmoCAB* encoding particulate methane monooxygenase (pMMO) and *xoxF* encoding a methanol dehydrogenase. No gene encoding a formaldehyde dehydrogenase was present and the formaldehyde to formate conversion follows the tetrahydromethanopterin (H_4_MPT) pathway. “*Ca.* Methylobacter favarea” B2 uses the Ribulose-Mono-Phosphate (RuMP) pathway for carbon fixation. Analysis of the MAG indicates that Na^+^/H^+^ antiporters and the urease system might be important in the maintenance of pH homeostasis of this strain to cope with acidic conditions. So far, thermoacidophilic *Methylobacter* species have not been isolated, however this study indicates that members of the genus *Methylobacter* can be found in distinct ecosystems and their presence is not restricted to freshwater or marine sediments.

**Supplementary Information:**

The online version contains supplementary material available at 10.1007/s10482-021-01525-7.

## Introduction

Volcanic and geothermal areas are hostile environments characterized by low pH, high temperature, geothermal gas emissions, and low O_2_ concentrations. One of the emitted geothermal gases is CH_4_, a potent greenhouse gas. Multiple studies have shown that aerobic methanotrophs may be important in reducing the emissions of geothermally produced CH_4_ (D'Alessandro et al. 2009; Etiope and Klusman 2002).

Phylogenetically, aerobic methanotrophs belong to the phyla Alphaproteobacteria, Gammaproteobacteria or Verrucomicrobia (Op den Camp et al. 2009). These methanotrophs are microorganisms that conserve energy by oxidizing CH_4_ to CO_2_, while using O_2_ as terminal electron acceptor (Hanson and Hanson 1996). The first step of the methane oxidation pathway involves the conversion of methane into methanol catalysed by a soluble or membrane-bound methane monooxygenase (Hanson and Hanson 1996). In the following steps, methanol is converted into CO_2_ via formaldehyde and formate using either lanthanide-dependent XoxF-type methanol dehydrogenase or a calcium-dependent MxaF-type methanol dehydrogenase (Keltjens et al. 2014). Carbon fixation occurs via the Ribulose-Mono-Phosphate (RuMP) pathway, the Serine Pathway or the Calvin–Benson–Bassham (CBB) cycle (Chistoserdova 2011; Khadem et al. 2011; Murrell 1992; Rasigraf et al. 2014; Sharp et al. 2014).

Previous studies using analysis of 16S rRNA genes or the diagnostic *pmoA* gene revealed the presence of methanotrophs in volcanic areas (Gagliano et al. 2014, 2016; Niemann et al. 2006). Thermoacidophilic methanotrophs from geothermal areas have been isolated and resulted in the first pure cultures of methanotrophic members of the phylum Verrucomicrobia (Dunfield et al. 2007; Erikstad et al. 2019; Islam et al. 2008; Pol et al. 2007; van Teeseling et al. 2014).

A recent metagenomic analysis of the volcanic soils of the Favara Grande, the main geothermal active area of Pantelleria Island, Italy, indicated the presence of a unique methanotrophic community, composed of Verrucomicrobia and Gammaproteobacteria (Picone et al. 2020). Different metagenome assembled genomes (MAGs) were retrieved. One of these MAGs was nearly complete and phylogenetic analysis showed that it represents the genome of a novel *Methylobacter* species. Typically, *Methylobacte*r species are found in freshwater sediments and wetland soils, where they account for a large fraction of aerobic methanotrophs (Smith et al. 2018). Thermoacidophilic *Methylobacter* species have, so far, not been isolated. In this study, we determined the phylogenetic position of this *Methylobacter,* and analysed the encoded metabolic potential.

## Materials and methods

### Sampling location and DNA isolation

Samples were collected at Favara Grande, Pantelleria, Italy 2017 (FAV1, 36° 50′ 80″ N; 11° 57′ 170″ E) and (FAV2, 36° 50′ 77″ N; 11°57′ 160″ E) during a field campaign in June 2017 (Picone et al. 2020). Soil samples (1–10, 10–15 and 12–20 cm depth) were taken using a core sampler (diameter 1.5 cm), stored in sterile 50 mL tubes and kept at 4 °C until DNA was extracted. In situ pH values were 4–4.5 with temperatures from 60 to 67 °C. Two different DNA extraction methods were used, namely Fast DNA Spin kit for soil (MP Biomedicals, Santa Ana, California), according to manufacturer’s instructions, and the CTAB method (Allen et al. 2006). DNA extraction was only successful from the FAV2 sampling site and the reads from the different depths and different extraction methods were combined for assembly and binning. For more detail see Picone et al. (2020).

### Genome sequencing, assembly and binning

The metagenome was sequenced on the Illumina sequencing platform. For library preparation the Nextera XT kit (Illumina, San Diego, California) was used according to the manufacturer’s instructions. Enzymatic tagmentation was performed starting with 1 ng of DNA, followed by incorporation of the indexed adapters and amplification of the library. After purification of the amplified library using AMPure XP beads (Beckman Coulter, Indianapolis), libraries were checked for quality and size distribution using the Agilent 2100 Bioanalyzer and the High sensitivity DNA kit. Quantitation of the library was performed by Qubit using the Qubit dsDNA HS Assay Kit (Thermo Fisher Scientific, Waltham, Massachusetts). The libraries were pooled, denatured and sequenced with the Illumina Miseq sequence machine (San Diego, California). Paired end sequencing of 2 × 300 base pairs was performed using the MiSeq Reagent Kit v3 (Illumina, San Diego, California) according the manufacturers protocol.

Reads were trimmed using BBDuk (BBMap), assembled by MEGAHIT v1.0.3 (Li et al. 2015) and binned using an in-house pipeline, using different binning algorithms, including BinSanity (Graham et al. 2017), COCACOLA (Lu et al. 2017), CONCOCT (Alneberg et al. 2014), MaxBin 2.0 (Wu et al. 2016), and MetaBAT 2 (Kang et al. 2019). DAS Tool 1.0 was used for consensus binning (Sieber et al. 2018) and CheckM was used to assess the MAG quality (Parks et al. 2015). The average nucleotide identity using BLAST (ANIb) is calculated using JSpeciesWS software with standard settings (Richter et al. 2016). An up-to-date Bacterial Core Gene (UBCG) phylogenetic tree was constructed using RAxML (Stamatakis 2014) on CIPRES Science Gateway V. 3.3 platform (Miller et al. 2012). PROKKA and the MicroScope platform were used to automatically annotate the draft genome (Seemann 2014; Vallenet et al. 2013) and genomic features were manually checked.

## Results and discussion

**Fig. 1:**
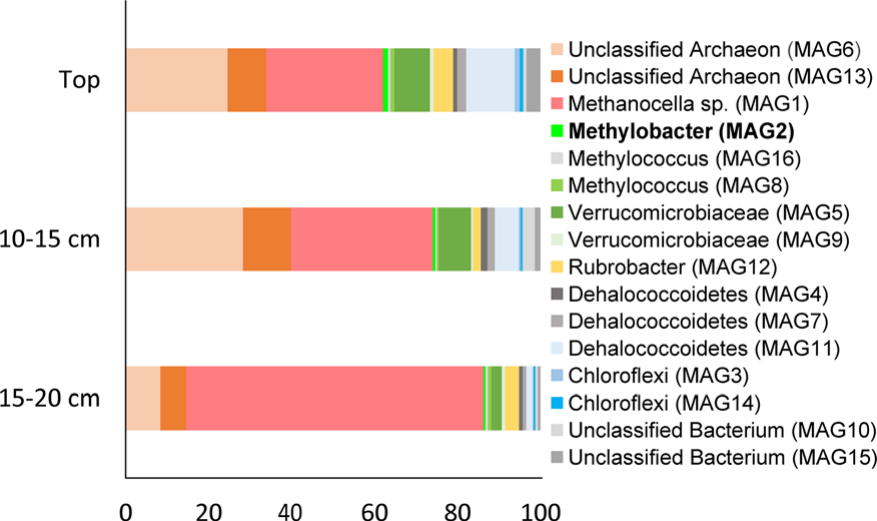
The relative abundance of the different binned MAGs (completeness > 95%) at the different depths of the geothermal soil of the Favara Grande, Pantelleria Island, Italy. The relative abundance of *Methylobacter* sp. B2 (MAG2) is 0.49%, 0.71% and 1.17% in the top layer, at 10–15 cm depth and at 15–20 cm depth, respectively

The Favara Grande is the main geothermal gas-emitting area on Pantelleria Island, Italy. The soil in this region is acidic, of high temperature and exposed to geothermal gas emission (D'Alessandro et al. 2009). It is devoid of any plant growth. At the FAV2 site the following physicochemical parameters were observed: temperatures 60–67 °C, pH 4–4.5, CH_4_ 1000–18,000 ppm and H_2_ 125–8400 ppm (see also Picone et al. 2020). CH_4_ and H_2_ concentrations were lowest close to soil surface, indicating active consumption of these gases. Metagenomic analysis of FAV2 soil samples revealed the presence of a diverse community of methanotrophs including those belonging to the phyla Verrucomicrobia and Gammaproteobacteria, at 6–11% and 2.5–3% relative abundance, respectively. No alphaproteobacterial methanotroph was detected (Picone et al. 2020) (Fig. 1). One of these FAV2 methanotrophs was classified as a *Methylobacter* species. The *Methylobacter* sp. B2 MAG was chosen for detailed analysis to achieve a better understanding of its metabolic potential and its relevance in the carbon cycle of geothermal soils. The MAG had a size of 4,086,539 bp, consisted of 134 contigs and had a GC content of 47.2%. CheckM analysis revealed that the completeness of this MAG was 99.1% with only 0.4% contamination. A total of 3955 genes could be identified, of which 3902 were protein coding genes and 53 were RNA genes. Functions could be assigned to 2164 protein coding genes (Table 1). Moreover, 88.4% of the predicted genes were assigned into Clusters of Orthologous Groups and these COG functional categories are compiled in Table 2.

**Table 1:** Genome statistics

Attribute	Value
Genome size (bp)	4,086,539
DNA coding (bp)	3,391,419
DNA G + C (%)	47.2
DNA scaffolds	134
Total genes	3955
Protein coding genes	3902
RNA genes	53
rRNA genes	3^a^
tRNA genes	36
Pseudo genes	11
Genes in internal clusters	–
Genes with function prediction	2164
Genes assigned to COGs	3338

^a^See supplementary material

**Table 2:** Number of genes associated with general COG functional category prediction

Code	Value	% of total^a^	Description
J	168	4.25	Translation, ribosomal structure and biogenesis
A	1	0.03	RNA processing and modification
K	149	3.77	Transcription
L	286	7.23	Replication, recombination and repair
B	2	0.05	Chromatin structure and dynamics
D	56	1.42	Cell cycle control, cell division, chromosome partitioning
V	67	1.69	Defense mechanisms
T	184	4.65	Signal transduction mechanisms
M	245	6.19	Cell wall/membrane/envelope biogenesis
N	98	2.48	Cell motility
U	114	2.88	Intracellular trafficking, secretion, and vesicular transport
O	158	3.99	Posttranslational modification, protein turnover, chaperones
C	210	5.31	Energy production and conversion
G	140	3.54	Carbohydrate transport and metabolism
E	209	5.28	Amino acid transport and metabolism
F	60	1.52	Nucleotide transport and metabolism
H	130	3.29	Coenzyme transport and metabolism
I	87	2.20	Lipid transport and metabolism
P	187	4.73	Inorganic ion transport and metabolism
Q	92	2.33	Secondary metabolites biosynthesis, transport and catabolism
R	416	10.52	General function prediction only
S	279	7.05	Function unknown
–	615	15.60	Not in COGs

^a^The total number is based on the number of protein coding genes (3902) in the genome

### Phylogeny

**Fig. 2:**
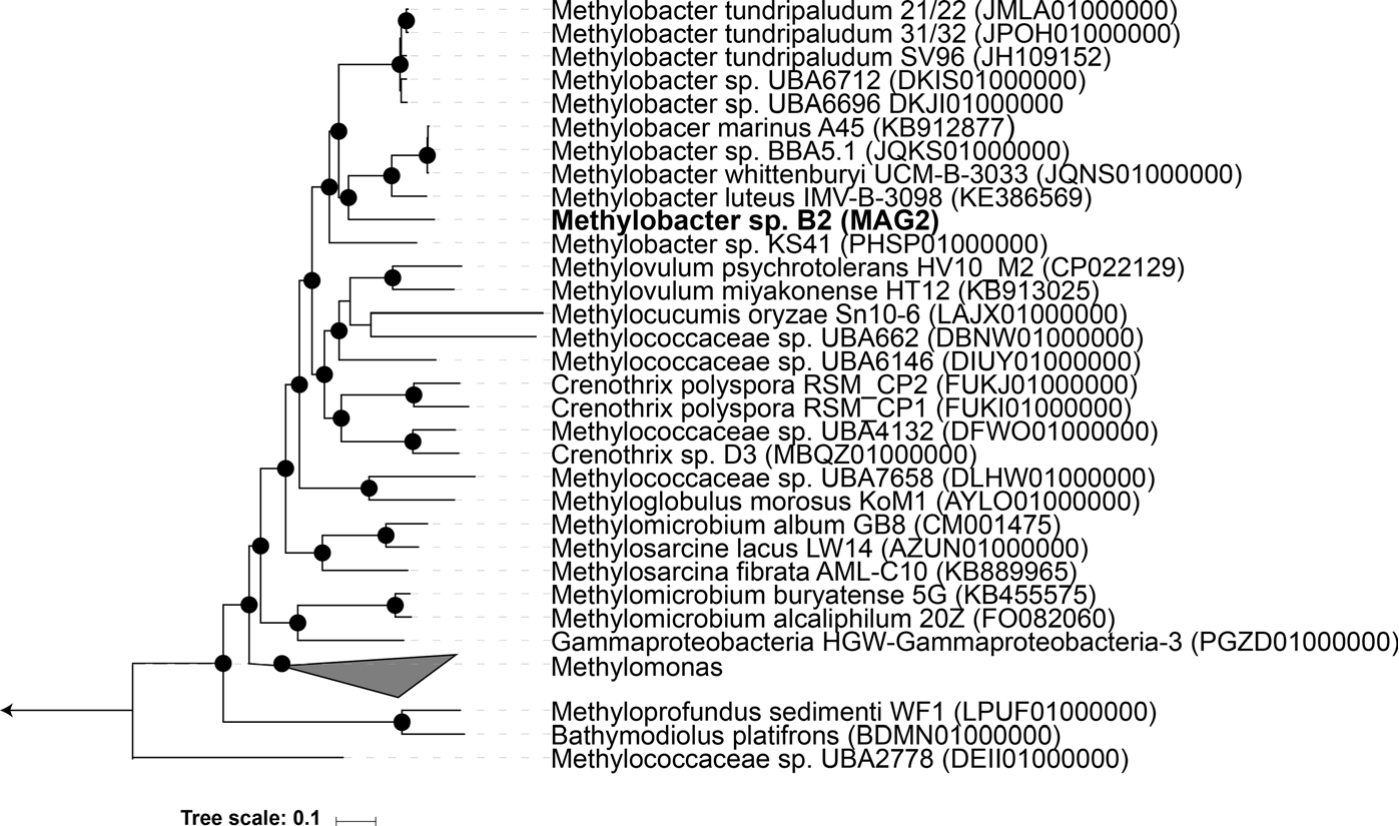
Up-to-date Bacterial Core Gene (UBCG) phylogenetic tree of MAG2 and members of the family *Methylomonaceae*, all with a genome completeness of > 90%. *Hyphomicrobium denitrificans* was used to root the tree, but removed from the tree for clarity. The tree was constructed using RAxmL. Bootstrap analysis was carried using 100 replications and percentage bootstrap values > 95% are indicated by black circles

The draft genome of *Methylobacter* sp. B2 contained one 16S rRNA gene. This gene is located at the end of a contig and the rest of the ribosomal RNA operon could not be detected. This often happens during binning due to high conservation of the rRNA gene sequences. We were able to assemble a contig with the full rRNA operon using all reads from the metagenome dataset and the contig with the 16S rRNA gene in MAG as a seed (Supplementary Material). Phylogenetic analysis of the 16S rRNA gene revealed that this gene clusters together with *Methylobacter* species (Supplementary Fig. S1, Kumar et al. 2016; Tamura 1992). The closest cultivated relative is *Methylobacter psychrophilus* Z-0021, showing a 16S rRNA gene identity of only 96.5%. Using a species boundary of 98.2%, this 16S rRNA identity indicated that the B2 MAG represented a novel species within the genus *Methylobacter*. Besides the 16S rRNA gene, *pmoA* is considered as molecular marker gene for defining methanotrophic taxa (Knief 2015). Phylogenetic analysis revealed of the *pmoA* gene in this MAG did not cluster with other *Methylobacter* species (Supplementary Fig. S2, Saitou and Nei 1987; Zuckerkandl and Pauling 1965). This is not uncommon as for *Methylobacte*r species, single-gene phylogenies resulted in inconsistencies, since this genus is assumed to be polyphyletic (i.e. having more than one common ancestor) (Orata et al. 2018). To circumvent this one-gene-polyphyletic classification problem, an UBCG (Up-to-date bacterial core gene set) phylogenetic tree was constructed. Rather than a single gene, this tree is based on 92 bacterial core genes (Na et al. 2018). The UBCG phylogenetic analysis showed that *Methylobacter* sp. B2 clusters within the *Methylobacter* genus (Fig. 2). Furthermore, average nucleotide identity (ANI) calculations gave values well below the threshold for species delimitation (95–96%), demonstrating that this MAG indeed represents a novel species within the genus *Methylobacter* (Table 3) (Chun et al. 2018), for which we propose the name “*Candidatus* Methylobacter favarea” B2.

**Table 3:** Average nucleotide identity (ANI) analysis of *Methylobacter* sp. B2 other representatives of this genus using JSpeciesWS software

Strain	Strain name	1	2	3	4	5	6	7	8	9
1	*Methylobacter* sp. B2 (MAG2)		73.7	75.9	75.9	72.8	74.6	74.1	75.9	73.3
2	*Methylobacter marinus* A45	73.4		74.7	74.6	94.6	72.6	84.3	74.7	96.1
3	*Methylobacter tundripaludum* SV96	75.5	74.4		95.4	70.6	75.4	74.7	95.6	74.2
4	*Methylobacter tundripaludum* 31/32	75.5	74.3	95.3		71.3	75.6	75.0	97.9	74.0
5	*Methylobacter* sp. BBA51	73.0	98.4	72.2	73.3		71.9	82.7	73.5	74.8
6	*Methylobacter* sp. KS41	74.8	73.0	76.1	76.1	72.6		73.4	76.2	72.4
7	*Methylobacter luteus*	73.9	84.5	75.0	75.1	79.7	73.1		75.2	83.6
8	*Methylobacter tundripaludum* 21/22	75.5	74.5	95.6	98.1	71.9	75.6	76.1		74.3
9	*Methylobacter whittenburyi*	73.3	98.2	74.6	74.6	80.3	72.2	84.8	74.6	

All reference genomes show a completeness of at least 90% and a contamination of maximum 5%

### Analysis of the encoded metabolic potential

#### Methanotrophy

**Fig. 3:**
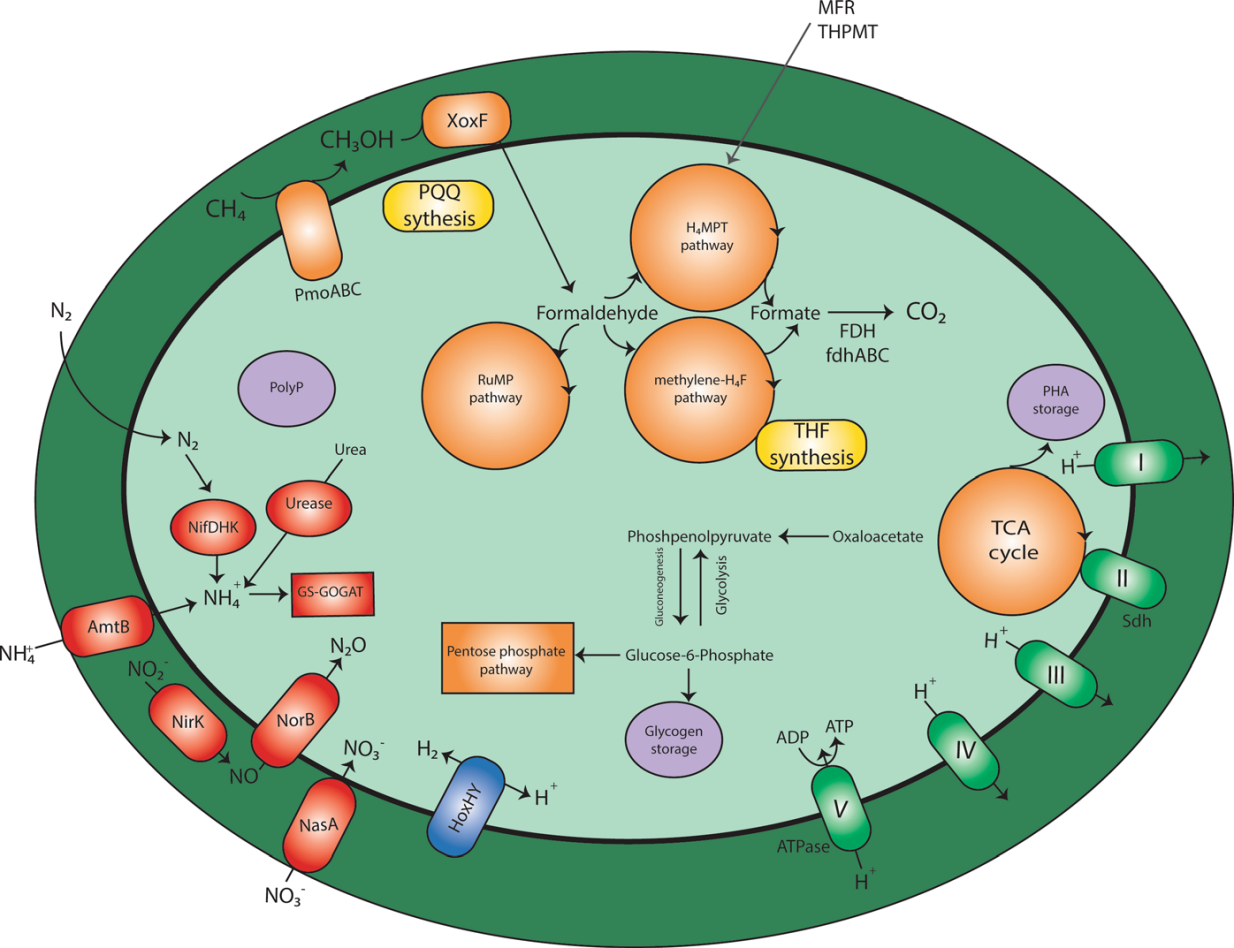
Cell metabolism of “*Ca. Methylobacter favarea*”* B2*. *Pmo* particulate methane monoxygenase, *XoxF* XoxF-type methanol dehydrogenase, *FDH* formate dehydrogenase, *NifDHK* nitrogenase, *Nirk* dissimilatory nitrite reductase, *NorB* nitric oxide reductase, *HoxHY* hydrogenase. Enzyme complexes of the electron transport chain are labeled by Roman numerals

The first step in the methane oxidation pathway is the conversion of methane into methanol catalyzed by a membrane-bound or soluble methane monooxygenase. Analysis of the draft genome (see Fig. 3 for a schematic representation) revelaed one *pmoCAB* gene cluster, encoding for the particulate membrane-bound, methane monooxygenase (pMMO). Typically, but not in all methanotrophs, the *pmoCAB* gene cluster is complemented with a *pmoD* gene. Recent studies show that *pmoD* is essential for pMMO activity, and probably involved in copper incorporation (Fisher et al. 2018). In draft genome of “*Ca.* Methylobacter favarea” B2, a homolog of the *pmoD* gene (METHB2_v1_630010) was present, but the gene is not located near the *pmoCAB* gene cluster. Genes encoding for the soluble methane monooxygenase (sMMO) were not detected.

“*Ca.* Methylobacter favarea” B2 catalyses the second step of the methane oxidation pathway, the conversion of methanol into formaldehyde, using a rare earth element (REE) and pyrroloquinoline quinone (PQQ) dependent XoxF-type methanol dehydrogenase (MDH) (Keltjens et al. 2014; Pol et al. 2014). In the genome, XoxF (METHB2_v1_30111), the catalytic MDH protein and XoxJ (METHB2_v1_30112), involved in protein binding for stability or electron transfer (Versantvoort et al. 2019), were encoded. The *xoxG* gene, which encodes for the cytochrome c_L_, that accepts electrons from MDH (Keltjens et al. 2014), could not be detected within the *xoxF/xoxJ* gene cluster. However, XoxF-MDH systems do not follow a general organizational pattern at genomic level (Keltjens et al. 2014) and this MAG contains several mono-heme cytochromes with the heme-binding motif CxxCH and the conserved methionine typical for XoxG proteins (METHB2_v1_70040, METHB2_v1_200048, METHB2_v1_230021, METHB2_v1_350009, METHB2_v1_350010 and METHB2_v1_510007). These cytochromes could serve as electron acceptor for XoxF (Versantvoort et al. 2019). During methanol oxidation, electrons are transferred to the cytochrome via the cofactor PQQ and all genes for the synthesis of PQQ were present in this MAG. No genes encoding for the calcium-dependent MxaF-MDH could be identified.

XoxF-MDH requires lanthanides as metal cofactor (Pol et al. 2014) but the mechanism of their uptake in bacterial cells in not completely clarified. Recently, the lanthanide binding protein Lanmodulin was identified and postulated to function as cargo protein for lanthanides from a lanthanide transporter to MDH (Cotruvo et al. 2018). Lanmodulin seems not to be encoded in the MAG. Other studies indicated that TonB-dependent transporters might be involved in REE transport over the outer membrane, which are encoded by *tonB* or *cirA* genes (Ochsner et al. 2019; Roszczenko-Jasińska et al. 2020). The draft genome of “*Ca.* Methylobacter favarea” B2 contains several *tonB* and *cirA* genes (METHB2_v1_20123, METHB2_v1_30066, METHB2_v1_130008, METHB2_v1_200030 and METHB2_v1_300037) whereby METHB2_v1_20123 shows the highest similarity (33% based on amino acid composition) with the putative REE transporter of *Methylorubrum extorquens* PA1. None of these transporters are located near the methanol dehydrogenase genes as in *Methylorubrum extorquens* PA1 (Ochsner et al. 2019).

In the next step of the CH_4_ oxidation pathway, formaldehyde is oxidized to formate. Formaldehyde is a key metabolite, since it is used for carbon assimilation by proteobacterial methanotrophs. Furthermore, formaldehyde oxidation is considered to be essential, since intracellular concentrations of toxic formaldehyde should remain low (Chistoserdova 2011). However, this MAG does not seem to contain a gene encoding for formaldehyde dehydrogenase, which prevents direct formaldehyde to formate conversion. In vitro studies showed that XoxF-MDH from *M. extorquens* AM1 and *M. fumariolicum* SolV can use formaldehyde as substrate leading to the hypothesis that XoxF-type MDHs oxidize methanol into formate (Pol et al. 2014; Good et al. 2018). In contrast, in vivo, the XoxF-type MDH of *M. extorquens* AM1 was shown to produce formaldehyde, which can be used for carbon assimilation (Good et al. 2018).

In *M. extorquens* AM1 the lanthanide-dependent ethanol dehydrogenase ExaF was involved in formaldehyde oxidation (Good et al. 2018). As there was no ethanol dehydrogenase nor a MxaF-MDH found in the draft genome of “*Ca.* Methylobacter favarea” B2 to oxidize formaldehyde, indicating that this strain possibly uses a different pathway for formaldehyde oxidation. The most widespread formaldehyde conversion pathway is the glutathione-linked formaldehyde oxidation pathway. The first step in this pathway is the reaction of formaldehyde with glutathione. The glutathione-dependent formaldehyde dehydrogenase enzyme accelerates this spontaneous reaction (Goenrich et al. 2002). This enzyme could be detected in the “*Ca.* Methylobacter favarea” B2 MAG (METHB2_v1_3500004). In the following two steps, formate is formed by glutathione-dependent formaldehyde dehydrogenase and S-formylglutathione hydrolase, however none of these enzymes could be detected within “*Ca.* Methylobacter favarea” B2.

Alternatively, formaldehyde can be converted to formate by the tetrahydromethanopterin (H_4_MPT) pathway or the methylene-tetrahydrofolate (methylene-H_4_F) pathway. The genes for both pathways were detected in draft genome of “*Ca.* Methylobacter favarea” B2, suggesting that this strain can use both pathways, as described for *Methylococcus capsulatus* Bath (Chistoserdova et al. 2005). Proteomics studies revealed that the H_4_MPT-pathway is used for formaldehyde to formate conversion in *M. capsulatus* Bath (Kao et al. 2004). Whether “*Ca.* Methylobacter favarea” B2 uses the H_4_MPT-pathway or the methylene-H_4_F-pathway remains uncertain. However, the genes for the synthesis of the cofactors methanofuran (MFR) and tetrahydromethanopterin (THPMT) were lacking, whereas the genes needed for the synthesis of tetrahydrofolate (THF) were present. This suggests that the H_4_MPT-pathway can only be used whenever the cofactors are supplied by other members of the microbial community.

Formate dehydrogenase catalyses the final step in the methane oxidation pathway, namely the conversion of formate into CO_2_. Typically, methanotrophs encode for multiple formate dehydrogenases (Chistoserdova 2011; Flynn et al., 2016). “*Ca.* Methylobacter favarea” B2 encodes for two different formate dehydrogenases, a NAD-dependent FDH encoded by a single gene (METHB2_v1_310032) and a molybdopterin containing formate dehydrogenase encoded by the three genes *fdhABC* (METHB2_v1_500021, METHB2_v1_500022 and METHB2_v1_500023).

#### Energy conservation and respiration

“*Ca.* Methylobacter favarea” B2 uses O_2_ as terminal electron acceptor. The NADH:ubiquinone reductase genes (complex I, *nuoABCDEFGHIJKLMN*) were found in the MAG, together with genes encoding the succinate dehydrogenase (complex II), cytochrome bc_1_ (complex III) and cytochrome-c-oxidase (complex IV) complexes. The proton motive force generated by the respiratory chain can be used by the ATP-generating ATPase (complex V).

#### Carbon fixation

“*Ca.* Methylobacter favarea” B2 uses the ribulose-mono-phosphate (RuMP) pathway for carbon fixation, as do other *Methylobacter* species (Flynn et al. 2016). Interestingly, a nearly complete serine pathway could also be detected in the genome of “*Ca.* Methylobacter favarea” B2. Usually, the serine pathway is used by alphaproteobacterial methanotrophs as carbon fixation pathway and gammaproteobacterial methanotrophs only contain parts of the serine pathway. Typically, gammaproteobacterial methanotrophs lack the enzyme phosphoenolpyruvate carboxylase (Chistoserdova 2011), however, the “*Ca.* Methylobacter favarea” B2 MAG encodes for every enzyme in the serine pathway except the serine-glyoxylate aminotransferase. In contrast to Verrucomicrobial methanotrophs (Khadem et al. 2011), the Calvin–Benson–Bassham cycle cannot be used for carbon fixation in “*Ca.* Methylobacter favarea” B2, since RuBisCO genes are lacking.

#### Alternative substrates

All genes for the glycolysis and gluconeogenesis were found and the TCA cycle genes were present. Genes of the glyoxylate shunt were partially detected, the gene encoding for isocitrate lyase is found in the draft genome, but a gene encoding for malate synthase could not be detected. The pentose phosphate pathway was present. Furthermore, transporters for a variety of organic molecules could be predicted. This indicates that “*Ca.* Methylobacter favarea” B2 could benefit from a mixotrophic lifestyle or survival modus. Furthermore, “*Ca.* Methylobacter favarea” B2 seemed to be able to store a variety of carbon compounds, including glycogen, polyhydroxybutyrate and polyphosphates.

The “*Ca.* Methylobacter favarea” B2 MAG contained an oxygen tolerant NAD-coupled hydrogenase, belonging to group 3d bidirectional hydrogenases (Greening et al. 2016). H_2_ is an abundant electron donor in the FAV2 soils (D'Alessandro et al. 2009; Gagliano et al. 2016; Picone et al. 2020) and simultaneous CH_4_ and H_2_ oxidation is reported for different methanotrophs (Chen and Yoch 1987; Hanczar et al. 2002; Mohammadi et al. 2017; Carere et al. 2017). However, it is unlikely that “*Ca.* Methylobacter favarea” B2 can grow as ‘Knallgas’ bacterium, since it requires formaldehyde for carbon fixation. A mechanism for CO_2_ fixation is not detected in this MAG.

#### Nitrogen

Ammonia can be used as nitrogen source and is assimilated using either the glutamate dehydrogenase (METHB2_v1_670013) or the glutamine synthetase (METHB2_v1_100031) and glutamate synthase (METHB2_v1_410017). Nitrate and nitrite could also serve as nitrogen source. The activity of the assimilatory nitrate reductase NasA (METHB2_v1_40058) and nitrite reductase NirBD (METHB2_v1_40055, METHB2_v1_40054) would results in the production of ammonium. Genes encoding urease (METHB2_v1_20024, METHB2_v1_20025 and METHB2_v1_20026) and nitrogenase (*nifHDK*, METHB2_v1_180032, METHB2_v1_180033 and METHB2_v1_180034) were present, indicating that urea and N_2_ could also be used as nitrogen source in this severely N-limited ecosystem. The genes encoding the nitrite reductases NirK (METHB2_v1_00392), NirS (METHB2_v1_02231) and nitric oxide reductase NorBC (METHB2_v1_01347, METHB2_v1_01348) were found, suggesting that “*Ca.* Methylobacter favarea” B2 may be capable of partial denitrification.

#### pH homeostasis

In order for “*Ca.* Methylobacter favarea” B2 to thrive in an acid environment, it is important to control the intracellular pH. The maintenance of pH homeostasis is a result of restriction of proton permeation, internal consumption of protons and enhancement of proton pumps (Guan and Liu 2020). ATPases can pump out electrons and release acid stress, but this requires ATP (Liu et al. 2015a). “*Ca.* Methylobacter favarea” B2 encodes for different Na^+^/H^+^ antiporters (METHB2_v1_30076, METHB2_v1_70105, METHB2_v1_150016 and METHB2_v1_840011), which might be important for proton exchange as well (Slonczewski et al. 2009).

There are different mechanisms on intracellular proton consumption, which generates alkaline products. Several microorganisms use an amino-acid tolerance system to decrease the intracellular pH. However, both the arginine deaminase (ADI) system and the glutamate-dependent acid tolerance system (Liu et al. 2015b; Reeve and Reid 2016; Shabayek and Spellerberg 2017) could not be detected in the draft genome of “*Ca.* Methylobacter favarea” B2 Instead, “*Ca.* Methylobacter favarea” B2 could use the urease system for proton consumption. Since the genome encodes a urease, this enzyme can transform urea into ammonia and CO_2_ at the expense of a proton, whereby it regulates the internal pH (Miller and Maier 2014). Interestingly, the urease genes are widespread amongst the geothermal microorganisms of the Favara Grande (Picone et al. 2020), indicating that this urease system might be an important mechanisms in pH homeostasis within geothermal microorganisms.

#### Ecological role

Typically, *Methylobacte*r species are found in freshwater oxic sediments, terrestrial habitats and marine ecosystems, where they account for a large fraction of aerobic methanotrophy (Hao et al., 2020; Khatri et al. 2019; Smith et al. 2018). Thermoacidophilic *Methylobacter* species have, so far, not been isolated. Thermophilic Gammaproteobacteria are found within the family *Methylothermaceae* and the genus *Methylocaldum* (Houghton et al. 2019) and not within the family *Methylomonaceae.* Previously, 16S rRNA gene amplicon sequencing and metagenomic sequencing revealed that *Methylobacter* sp. are abundant methanotrophs in the geothermal soils of the Favara Grande (Gagliano et al. 2016). Other geothermal soil microbial communities, such as the one in the Solfatara Crater near Naples, Italy, did not show the presence of *Methylobacter* species (Crognale et al., 2018), indicating that we still have to learn more about the metabolic diversity of this important group of methanotrophs.

## Supplementary Information

Below is the link to the electronic supplementary material.

Supplementary file 1 (PDF 242 KB)

## Data Availability

DNA sequences (raw sequence reads and MAGs) have been deposited in NCBI BioProject database with accession number PRJEB36447. The draft genome is available at NCBI under Accession No. GCA_902806695.
