# Supplementary material for: Draft genome of a novel methanotrophic Methylobacter sp. from the volcanic soils of Pantelleria Island
Source: Antonie Van Leeuwenhoek. 2021 Feb 10;114(3):313–24. doi: 10.1007/s10482-021-01525-7 (PMC7902576; doi:10.1007/s10482-021-01525-7)
Supplement: Supplementary file 1 — (PDF 242 KB) [file 10482_2021_1525_MOESM1_ESM.pdf]

Antonie van Leeuwenhoek

Supplementary Material

Draft genome of a novel methanotrophic *Methylobacter* sp. from the volcanic soils of Pantelleria Island

Carmen Hogendoorn[1], Nunzia Picone[1], Femke van Hout[1], Sophie Vijverberg[1], Lianna Poghosyan[1], Theo A. van Alen[1], Jeroen Frank[1], Arjan Pol[1], Antonia L. Gagliano[2], Mike S.M. Jetten[1], Walter D'Alessandro[2], Paola Quatrini[3] & Huub J.M. Op den Camp[1*]

[1] Department of Microbiology, IWWR, Radboud University, Heyendaalseweg 135, 6525 AJ Nijmegen, Netherlands

[2] Istituto Nazionale di Geofisica e Vulcanologia, Sezione di Palerma, Via U. La Malfa 153, 90146 Palermo, Italy

[3] Department of Biological, Chemical and Pharmaceutical Sciences and Technologies (STEBICEF), University of Palermo, Viale delle Scienze Ed. 16, 90128 Palermo, Italy

*Address correspondence to: Huub J.M. Op den Camp, h.opdencamp@science.ru.nl, +31 243652657

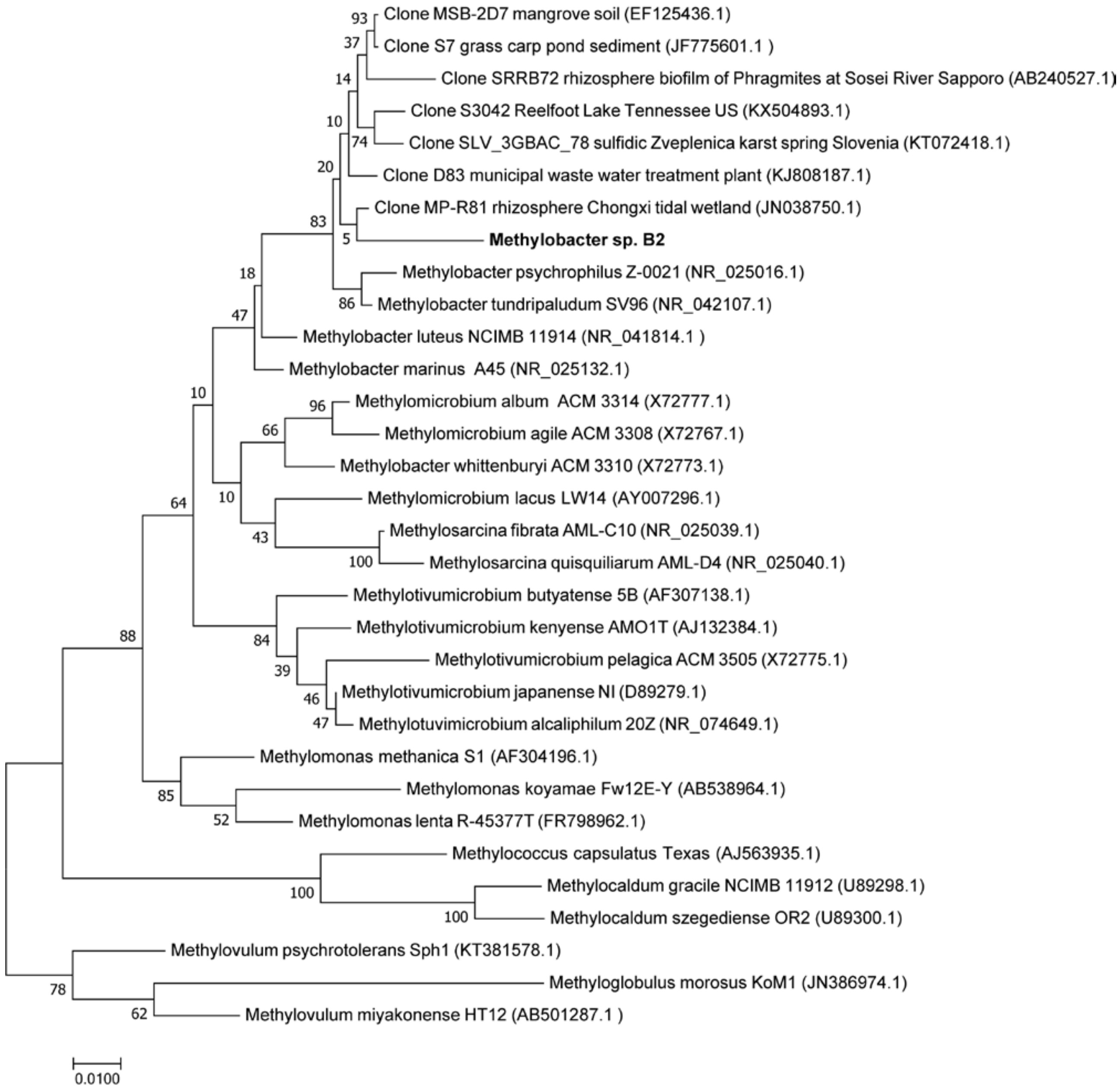

Supplementary Fig. S1 Phylogenetic tree of binned MAG2 and member of the members of the family *Methylomonaceae* based on 16S rRNA gene sequences. The three was constructed using the Maximum Likelihood method bases on the Tamura-Nei model (Tamura, 1992). Bootstrap percentage values (1000 replicates) are given at each node and the analysis was performed in Mega7 (Kumar et al., 2016).

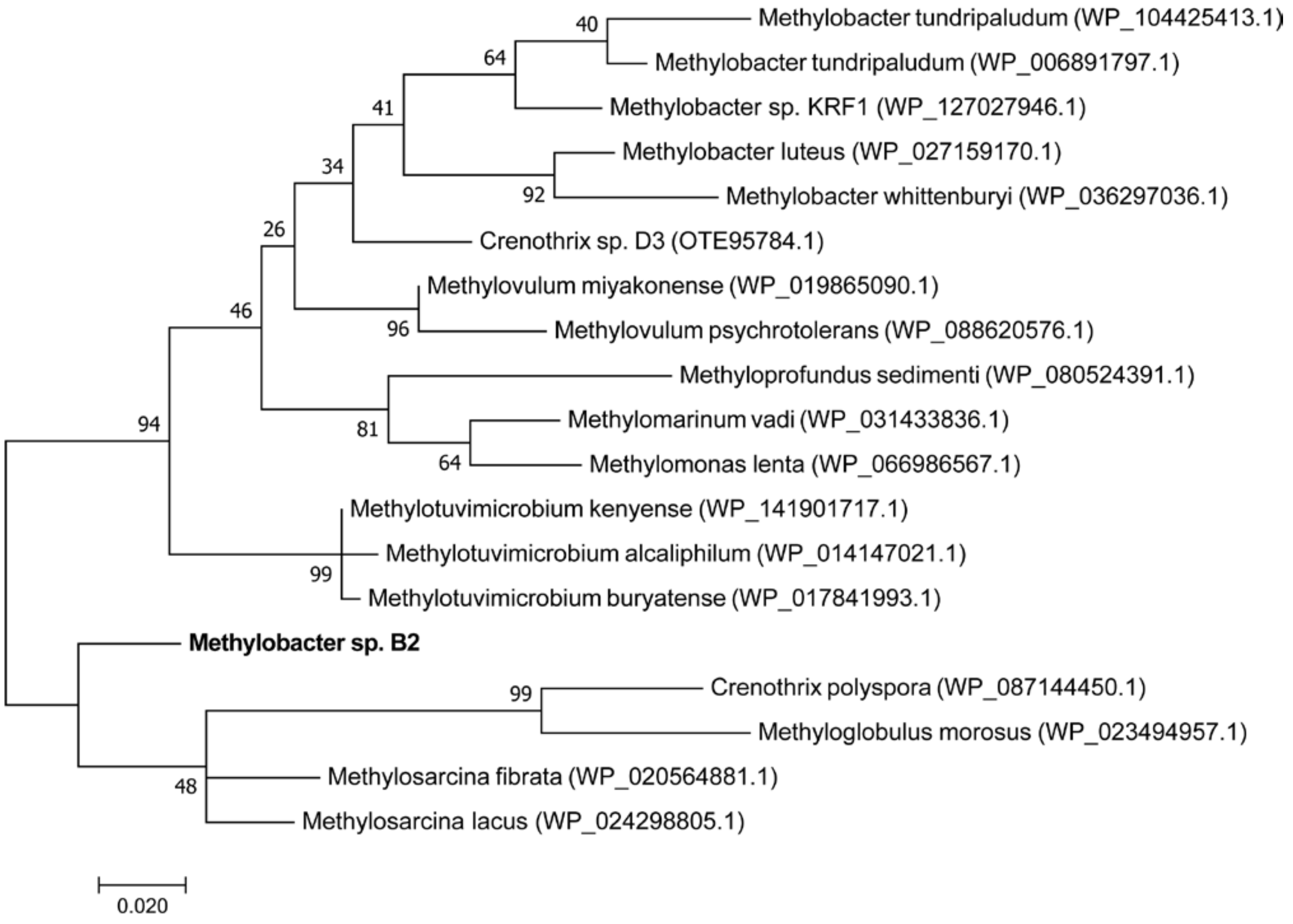

Supplementary Fig. S2 Phylogentic tree of PmoA protein sequences of members of the family *Methylomonaceae*. The three was constructed using the Maximum Likelihood method (Saitou and Nei, 1987). Bootstrap percentage values (1000 replicates) are given at each node. Evolutionary distances were calculated using the Poission correction method (Zuckerkandl and Pauling, 1965) and the analysis was performed in MEGA7 (Kumar et al., 2016).

```
LOCUS       NODE_5474    5769 bp   coverage 21.3x   DNA linear
DEFINITION  “Candidatus Methylobacter favarea” B2, rRNA contig

FEATURES    Location/Qualifiers
     source 1..5769
            /organism="Candidatus Methylobacter favarea"
            /strain="B2"
            /isolation_source="Pantelleria volcanic soil"
rRNA        443..1966
            /locus_tag="METHB2_R0001"
            /product="16S ribosomal RNA"
tRNA        2041..2114
            /locus_tag="METHB2_R0002"
            /product="tRNA-Ile"
tRNA        2170..2242
            /locus_tag="METHB2_R0003"
            /product="tRNA-Ala"
rRNA        2485..5375
            /locus_tag="METHB2_R0004"
            /product="23S ribosomal RNA"
rRNA        5543..5658
            /locus_tag="METHB2_R0005"
            /product="5S ribosomal RNA”
ORIGIN
   1 cacgcattca ggcaatgttt gtacatcata gataaatgca ctagtacgat cggctgggcg
  61 gcggggtggg caaaaaaaac ccaagcattt caggcttggg tggtaatagg cgcttggcaa
 121 ttccctactt tcgcatggca acctgccaca ctatcatcgg cgctaagcgg tttcactgcc
 181 gagttcggga tgggatcggg tgggtcacgc tcgctatggt caccaagcaa actggtgtgg
 241 tcgacgttgc tctgtcgacc ttcggcagga catccgtcct ggccttggaa atctgtaatt
 301 cggatggctt gtggttttca tgcagataaa gcctcagccg gctaagctgg ggacctagcc
 361 tttttaacac ttgctcaaac tgattgggtg ttatatggtc aagcctcacg ggcaattagt
 421 acgggttagc tgcatgcatt actgcacttc cacatcccgc ctatcaacgt cgtagtctgc
 481 gacggccctt caggggactt aaagtcccgg tgagatctca tcttgggagg ggcttcccgc
 541 ttagatgctt tcagcggtta tcctgtccga acatagctac ccggcaatgc cattggcatg
 601 acaaccggaa caccagaggt tcgtccactc cggtcctctc gtactaggag cagcttccct
 661 caaatctcaa acgcccacgg cagataggga ccgaactgtc tcacgacgtt ctgaacccag
 721 ctcgcgtacc actttaaatg gcgaacagcc ataccсttgg gacctgcttc agccccagga
 781 tgtgatgagc cgacatcgag gtgccaaaca ccgccgtcga tatgaactct tgggcggtat
 841 cagcctgtta tccccggagt acctttatc cgttgagcga tggcccttcc attcagaacc
 901 accggatcac tatgacctac tttcgtacct gctcgatgtg tctatctcgc agtcaagcac
 961 ccttatgcca ttgcactcat tgcctgattt ccgaccaggc tgagggtacc ttcgtgctcc
1021 tccgttactc tttaggagga gaccgcccca gtcaaactac ccaccagaca ctgtccccaa
1081 tccggataac ggatccaggt tagaacttca aatcaaccag ggtggtattt caagggtggc
1141 tccacagcaa ctggcgttgc cgcttcacag cctcccacct atcctacaca agttgagtca
1201 aagtccagtg tcaagctata gtaaaggttc acggggtctt tccgtctagc cgcgggtata
1261 cggcatcttc accgcaattt caatttcact gagtctcggg tggagacagt gtggccatcg
1321 ttacgccatt cgtgcaggtc ggaacttacc cgacaaggaa tttcgctacc ttaggaccgt
1381 tatagttacg gccgccgttt actggggctt cgatcaagag cttcgcttac gctaacccca
1441 tcaattaacc ttccagcacc gggcaggcgt cacaccctat acgtcctctt tcgagtttgc
1501 agagtgctat gtttttgcta aacagtcgca gccaccaatt tattgcaacc ctcttgggct
1561 ccatgagcaa gtcacttcac ctaccaaggg tgtaccttct cccgaagtta cggtaccatt
1621 ttgcctagtt ccttcacccg agttctctca agcgccttgg aattctcatc ccacccacct
1681 gtgtcggttt ggggtacggc cactgataac ctgaagctta gaggtttttc ttggaagctt
1741 ggcatcaatc acttcgcagc tccgaagagc cgctcgtcat cacgtctcag aatatagggt
1801 cccggatttg cctaagaccc ctccctacac gcttaaactg ccacatccaa ccgacagctg
1861 atctagcctt ctccgtcacc ccatcgcagt tatcaccggt acaggactat taacctgttt
1921 gccatcgact acgcctttcg gcctcgcctt aggtaccgac taaccctgcg tcgattaacg
1981 ttgcgcagga aaccttgggt tttcggcgtg agggtttttc acccccatta tcgttactca
2041 tgtcagcatt cgcacttctg atacctccag caaacttctc agttcacctt cgcaggcgta
2101 cagaacgctc ctctaccacc acacctaagt gtgatccgta gcttcggtac taggcttagc
2161 cccggtaaat cttccgcgca gaccgactcg accagtgagc tattacgctt tctttaaagg
2221 atggctgctt ctaagccaac ctcctggctg tctgggcctt tccacatcgt ttcccactga
2281 gcctagattt ggggacctta gctgacggtc tgggctgttt cccttttcac gacggacctt
2341 atcacccgcc gtgtgtctcc cgtgctgaca cttgttggta ttcggagttt gcatcgggtt
2401 ggtaagtcgg gatgaccccc tagccgaaac agtgctctac ccccaacagt gatacacgag
2461 gcgctaccta aatagctttc gaggagaacc agctatctcc gagcttgatt agcctttcac
2521 tccgatccac agctcatccc cgcatttttc aacatacgtg ggttcggccc tccagttagt
```

2581 attacccaac cttcagcctg gccatggata gatcgcccgg tttcgggtct aatcccagcg
2641 actgaacgcc ctgttaagac tcgctttcgc tacgcctccc ctagtcggtt aagcttgcca
2701 ctgagattaa gtcgctgacc cattatacaa aaggtacgca gtcacccttt aaggggggctt
2761 ccactgcttg tacgcatacg gtttcaggat ctatttcact ccgctctccg cggttctttt
2821 cgcctttccc tcacggtact ggttcactat cggtcagtaa ggagtattta gccttggagg
2881 atggtccccc cgtattcagt caacgtttca cgtgcgtcga cctactcgat ttcacctcta
2941 tgcagttttc gtgtacgggg ctatcacccg gtatcgccag actttccaga ctgttccact
3001 aacggcataa agacttaagg gctaatcccc gttcgctcgc cgctacttgg ggaatctcgg
3061 ttgatttctt ttcctccggg tacttagatg tttcagttcc ccgggttcgc ttcctgcagc
3121 tatgtattca ctgcacgatg accagctgat gctggccggg tttccccatt cggatatccg
3181 cggatcaggg ttagtttgca aacttcccgc ggcttttcgc atgctacaac gtccttcatc
3241 gcctcttact gcctaggcat ccaccgtatg cgcttattca cttgaccata taaccccaat
3301 aagtctggcg ttatacggat cagctgatat tttcactttt tctgcttgaa acgtattctt
3361 gctgatcgtt atggttacaa ccaatccgct tgaattcgtt agccacatcc cttgcctctg
3421 ttgccagagc tttgggatag acttgttaca gatttccaca ttgttaaaga gctatcagta
3481 attgcttacc aatgctataa aacctgagac aagtcttata gcattggtgg agccagggag
3541 gatcgaactc ccgacctcct gcgtgcaagg caggcgctct cccagctgag ctatggcccc
3601 agatgttcca ggttccctag cccgttcacc gcttttacgt gtccgtaaaa agtggtgggc
3661 ctgggaggat ttgaacctcc gacctcaccc ttatcagggg tgcgctctaa ccaactgagc
3721 tacaggcccc aggctcatcc tgcgggtgat caaaataatt tgttgtggat actcatgaca
3781 acagtcagct gcaatttgta aggaggtgat ccagccccag gttcccctag ggctaccttg
3841 ttacgacttc accccagtca tgaatcacaa agtggtgagc gccctcctga aaggttagac
3901 tacccacttc ttttgcaacc cactcccatg gtgtgacggg cggtgtgtac aaggcccggg
3961 aacgtattca ccgcgacatt ctgattcgcg attactagcg attccgactt catggagtcg
4021 agttgcagac tccaatccgg actaggaccg gctttctggg atttgcttga cttcgcagtt
4081 tcgcagccct ctgtaccggc cattgtagca cgtgtgtagc cctacccata agggccatga
4141 tgacttgacg tcgtccccac cttcctccgg tttatcaccg gcagtctccc tagagttccc
4201 gcctcgacgc gctggcaact aggaataagg gttgcgctcg ttacgggact taacccaaca
4261 tctcacgaca cgagctgacg acagccatgc agcacctgtc tctgagctcc cgaaggcact
4321 ccgctatctc tagcagattc tcaggatgtc aagggtaggt aaggttcttc gcgttgcatc
4381 gaattaaacc acatgctcca ccgcttgtgc gggcccccgt caattcattt gagttttaac
4441 cttgcggccg tactccccag gcggtcaact taaggcgtta gctgcgccac taagcctgta
4501 tataggccca acggctagtt gacatcgttt acggcgtgga ctaccagggt atctaatcct
4561 gtttgctacc cacgctttcg tacctcagcg tcagtttgag tccagggagt cgccttcgcc
4621 actggtgttc cttcagatct ctacgcattt caccgctaca cctgaaattc cactcccctc
4681 tactcaactc gagtacgcca gtttcaaatg ccgttcccgg gttaagcccg gggctttcac
4741 atctgactta acgcaccgcc tacgcacgct ttacgcccag taattccgat taacgctcgc
4801 accctccgta ttaccgcggc tgctggcacg gagttagccg gtgcttcttc tataggtaat
4861 gtcaagctgc cgggtattga ccggcaggtt ttcctcccca ttgaaagtgc tttacaaccc
4921 tcaggccttc ttcacacacg cggtattgct ggatcaggct tgcgcccatt gtccaatatt
4981 ccccactgct gcctcccgta ggagtctggg ccgtgtctca gtcccagtgt ggctgatcgt
5041 cctctcagac cagctataga tcgttgcctt ggtaggcctt taccccacca accagctaat
5101 ctaacgcagg ctcatctgat agcgaaaggt ccaaagatcc cctcctttcc cccgtagggc
5161 gtatgcggta ttagcgtggg tttccccacg ttgtccccca ctatcaggca gattcctacg
5221 cgttactcac ccgtccgcca ctcgtcagcg cccgaaggcc tgttaccgtt cgacttgcat
5281 gtgttaagca taccgccagc gttcaatctg agccatgatc aaactcttca gtttatatca
5341 ttttaccact caataagatg agtgaccaat cttggctcga ccaacaactc aacgtcattt
5401 acatcgtgaa ttaacgtgtg ttcttgctcg aataattatc gcaatgctaa actagtcgcc
5461 acaagtaccc acacaaatta ttttgatcag gtttttaaag agcatggagc cagtacccct
5521 tgagtcccgc aattatacag gacctattat atctgtcaag cactgccggg ctttaatttt
5581 tccatcccgc cctccccgga caattccctt gccccttcac aagccggcta ttaccccgca
5641 cttccttgtt acctggcaat accctgactt gaagcaccag taaaattacc ttcctcacag
5701 acaatcagcc atgaaagaac tgcgcattat acatgctcac catcaaccgt caacagttat
5761 ttttactcg
//
